# Hydrophobic Modification of Poly(γ-glutamic acid) by Grafting 4-Phenyl-butyl Side Groups for the Encapsulation and Release of Doxorubicin

**DOI:** 10.3390/pharmaceutics15051377

**Published:** 2023-04-29

**Authors:** Porochista Dorost, Montserrat García-Alvarez, Antxon Martínez de Ilarduya

**Affiliations:** Departament d’Enginyeria Química, Universitat Politècnica de Catalunya, ETSEIB, Diagonal 647, 08028 Barcelona, Spain

**Keywords:** poly(γ-glutamic acid), biodegradable nanoparticles, drug delivery nanoparticles, doxorubicin, drug encapsulation, pH-responsive drug delivery

## Abstract

The delivery of drugs is a great challenge, since most of active pharmaceutical ingredients developed today are hydrophobic and poorly water soluble. From this perspective, drug encapsulation on biodegradable and biocompatible polymers can surpass this problem. Poly(γ-glutamic acid) (PGGA), a bioedible and biocompatible polymer has been chosen for this purpose. Carboxylic side groups of PGGA have been partially esterified with 4-phenyl-butyl bromide, producing a series of aliphatic–aromatic ester derivatives with different hydrophilic–lipophilic balances. Using nanoprecipitation or emulsion/evaporation methods, these copolymers were self-assembled in a water solution, forming nanoparticles with average diameters between 89 and 374 nm and zeta potential values between −13.1 and −49.5 mV. The hydrophobic core containing 4-phenyl-butyl side groups was used for the encapsulation of an anticancer drug, such as Doxorubicin (DOX). The highest encapsulation efficiency was reached for a copolymer derived from PGGA, with a 46 mol% degree of esterification. Drug release studies carried out for 5 days at different pHs (4.2 and 7.4) indicated that DOX was released faster at pH 4.2, revealing the potential of these nanoparticles as chemotherapy agents.

## 1. Introduction

Cancer is one of the most lethal diseases these days [1]. The traditional treatment is chemotherapy, in which the anticancer drug is administered through an intravenous injection. The concentration of the drug in the systemic circulation after injection is initially high and subsequently decreases very fast due to hepatic and renal clearances, reducing its therapeutic effect [2]. On the other hand, most anticancer drugs have a low therapeutic window, which causes toxicity in several healthy tissues [3,4]. This problem can be fixed if the drug is administered in a controlled manner through a sustained release on the damaged tissue, using polymers as release regulators [5,6,7].

In the last few decades, scientists have been carrying out many studies for developing new drug delivery systems (DDS) that are able to optimize drug loading and release with a greater long life and effectiveness. Particularly, in the biomedical field, self-assembled systems made of biodegradable amphiphilic polymers at the nanoscale size, such as nanoparticles, polymer micelles, nanotubes, nanogels, and polymersomes, have received a lot of attention [8,9,10]. From this perspective, self-assembled coronal graft or block copolymer nanospheres are appealing systems [11,12]. These systems have the ability to self-assemble in aqueous media, forming nanostructured particles. The preparation and development of these structures are among the issues that biomedical sciences are facing in the field of DDS [13].

A great challenge is the optimization of polymer formulations, not only to improve the encapsulation efficiency of drugs, but also to reduce their toxicity and prolong their release [14,15,16]. A great advantage of using these nanostructures as chemotherapeutic DDS is based on the EPR (enhanced permeability and retention) effect, which is the mechanism in which small-size nanoparticles can extravasate the leaky vascularized vessels that are present in the tumors, and stay there due to the poor lymphatic drainage [17,18]. The hydrophilic shell of these nanoparticles prevents the interaction with plasma proteins, as well as reduces the uptake of macrophage cells. To prevent quick renal excretion and clearance by the reticuloendothelial system (RES), the polymeric nanoparticles must have an appropriate size [19,20].

Polymeric nanoparticles are usually obtained from block copolymers made of hydrophilic blocks, such as poly(ethylene glycol), and hydrophobic blocks, such as aliphatic polyesters (PLA, PGLA, PCL) [21,22]. Moreover, the chemical modification of hydrophilic polymers, such as polysacharides, polypeptides or poly(acrylic acid), with hydrophobic substituents are good alternatives for obtaining amphiphlic copolymers [23,24,25,26]. In this work, we have chosen this last method, using a microbial edible biopolymer poly(γ-glutamic acid) as the starting polymer [27,28].

PGGA is a poly(γ-peptide) produced by the fermentation of different bacterial strains, and it has been shown to be biodegradable, biocompatible and non-immunogenic [29]. It is a nylon-4 derivative with a carboxylic group attached to the γ-CH carbon. The relatively easy and direct chemical modification of their lateral carboxyl groups allows for tuning properties such as degradation and solubility rates, insertion of targeting molecules, stability, and the release encapsulated drugs [30,31].

In this work, the carboxylic groups of PGGA have been esterified with 4-phenyl-butyl bromide, providing a series of copolymers with different amphiphilic properties. A whole series of copolymers, with compositions ranging from 95/5 to 3/97, were obtained, with the aim of studying the effect of composition on both the self-assembly capacity to form nanoparticles and the encapsulation capacity of a hydrophobic drug. It was observed that these copolymers were able to self-assemble in an aqueous medium, forming nanospheres that have the ability to trap hydrophobic drugs, such as Doxorubicin (DOX). Since both PGGA and 4-phenyl-1-butanol are biocompatible [26,32], it is expected that the nanoparticles obtained in this work would maintain this biocompatibility.

## 2. Materials and Methods

### 2.1. Materials

Poly(γ-glutamic acid) (PGGA), supplied by Dr. Kubota of Meiji Co. (Tokyo, Japan), was used in this work. Dimethyl sulfoxide (DMSO) (99.9%), sodium hydrogen carbonate (NaHCO_3_) (>99.0%), citric acid monohydrate (C_6_H_8_O_7_·H_2_O) (>99.5%), and dichloromethane (DCM) (99.9%) were acquired from Lab Kem (St. Migjorn, Barcelona, Spain). The 4-Phenyl-butyl bromide (C_10_H_13_Br) (95.0%) and doxorubicin hydrochloride (DOX·HCl) (98%) were purchased from Sigma-Aldrich (St. Louis, MO, USA). Hexafluoroisopropanol (HFIP) was supplied by Apollo Scientific (Manchester, UK). Triethylamine (TEA) (99.5%), potassium chloride (KCl) (99.0%), sodium chloride (NaCl) (98.0%), and N-methyl-2-pyrrolidone (NMP) (99.0%) were provided by Panreac Química SLU (St. Garraf, Barcelona, Spain). Potassium dihydrogen phosphate (KDP) (99.5%) was obtained from Merck. Additionally, disodium hydrogen phosphate dodecahydrate (Na_2_HPO_4_ 12H_2_O) (>99%) was purchased from Scharlau (St. Gato Pérez, Barcelona, Spain), and poly(vinyl alcohol) (PVA) (Approx. M_w_ 3000) was supplied by Scientific Polymer Products, Inc. The dialysis membrane, with a molecular weight cut-off MWCO 6–8 kDa, was provided by Spectrum Labs (St. Broadwick, Compton, CA, USA).

### 2.2. Characterization

The ^1^H NMR spectra were recorded on a Bruker AMX-300 spectrometer (Billerica, MA, USA) at 25 °C. The ^1^H NMR spectra were acquired at 300.1 MHz. Samples were dissolved in deuterated chloroform (CDCl_3_) or DMSO-d_6_, and the spectra were internally referenced against tetramethylsilane (TMS). Of the sample, 10 mg was dissolved in approximately 1 mL of solvent for ^1^H NMR.

Fourier transform infrared spectra (FT-IR) were acquired using a Perkin-Elmer Frontier FT-IR spectrometer (Waltham, MA, USA), provided with a universal-attenuated total reflectance ATR accessory. Infrared spectra were recorded in the 4000–650 cm^−1^ range at a resolution of 4 cm^−1^, and 16 scans were collected.

Molecular weights were determined by GPC, using HFIP containing sodium trifluoroacetate (6.8 g·L^−1^) within Waters equipment (Foster City, CA, USA), provided with RI and UV detectors and HR5E and HR2 Waters linear Styragel columns (7.8 mm × 300 mm). Of the sample solution, 0.1 mL (0.1% *w*/*v*) was injected and chromatographed with a flow rate of 0.5 mL·min^−1^. The molar mass averages and distributions were calibrated against PMMA standards.

Thermogravimetric analyses were carried out under a nitrogen flow rate of 20 mL·min^−1^ and at a heating rate of 10 °C·min^−1^, within a temperature range of 30 to 600 °C, using a Mettler Toledo TGA2 (Columbus, OH, USA). Sample weights of around 5–10 mg were used in these experiments.

Dynamic light scattering studies were performed using a Zetasizer Nano ZS series Malvern instrument (Worcestershire, UK), equipped with a 4 mW He–Ne laser operated at a wavelength of 633 nm. The samples were placed in disposable cuvettes thermostated at 25 °C. The non-invasive back-scatter optical arrangement was used to collect the light scattered by the particles at an angle of 173°. The particle hydrodynamic sizes and ζ-potential measurements were examined.

Absorbance measurements were examined using a UV-visible spectrophotometer (Cambridge, England, UK) and the samples were dissolved in DMSO. The drug concentration was calculated with a calibration curve obtained from the known amounts of free DOX as standards. SEM images were taken with a field-emission JEOL JSM-7001F (JEOL, Tokyo, Japan) from platinum/palladium-coated samples. The samples were prepared by depositing a drop of the nanoparticle dialysis solution onto a copper surface. Different dilutions were essayed to observe free individual nanoparticles and DOX-encapsulated nanoparticles. The mean diameter of the nanoparticles was determined using the ImageJ software [33].

### 2.3. Esterification of PGGA

PGGA was esterified with 4-phenyl-butyl bromide in solution, using a general procedure reported by Kubota et al. [34]. Specifically, 500 mg (4.0 mmol) PGGA was dissolved in 100 mL of NMP and left under stirring at 80 °C for 1 h, for the complete dissolution of the polymer. Afterwards the solution was cooled down to 60 °C and variable amounts of NaHCO_3_ were added to the solution, depending on the degree of esterification. After that, 4-phenyl-butyl bromide was slowly added at a necessary amount, to reach the desired conversion. The reaction was left to proceed for 48 h, until no evolution was observed in the reaction and the esterified polymer was recovered by precipitation in acidic water. Then, the copolymer was washed with neutral water and dried under vacuum for 24 h. The copolymers obtained were named PGGAH_x_PhB_y_, x and y being the molar ratio (%) of unmodified and modified repeating units.

### 2.4. Nanoparticle Preparation

Two different methods were assayed to prepare the nanoparticles: (1) dialysis/precipitation (nanoprecipitation) and (2) emulsion/evaporation (nanoemulsion). In the first method, 5 mg of the copolymer was dissolved in 1 mL of NMP, and afterward, 1 mL of distilled water was added dropwise under magnetic stirring. The solution was introduced in a dialysis bag of cellulose, with a molecular weight cut-off of 6000–8000 Da, and was dialyzed for 24 h at room temperature. Distilled water was replaced four times at 2, 5, 9, and 17 h, to remove any residual NMP solvent. The second emulsion/evaporation method was also assayed for copolymers with higher degrees of esterification. Briefly, 5 mg of copolymer was dissolved in 1 mL DCM, and this solution was added to 10 mL of 0.5% poly(vinyl alcohol) (PVA) aqueous solution. The mixture was emulsified with the help of an ultrasounds bath for 45 s (three times). Then, this solution was dispersed in 20 mL of water under magnetic stirring and DCM was rotary-evaporated. Particle average diameters, distributions and surface charges of nanoparticles were determined by DLS.

### 2.5. Stability of Nanoparticles in Solution

After producing nanoparticles, they were kept in solution at 2–4 °C for 4 weeks. The effect of storage time on the stability of the dispersion was evaluated.

### 2.6. Doxorubicin Loading and Releasing

Doxorubicin hydrochloride (DOX·HCl) was used as a drug model in this study. Of the DOX·HCl, 2 mg was dissolved in 2 mL of DMSO, and then 20 μL of TEA was added, leaving the solution for 24 h under magnetic stirring in a dark room at room temperature. TEA was added in order to remove the HCl from the DOX salt, enhancing drug encapsulation [35]. On the other hand, the PGGAH_x_PhB_y_ copolymer (10 mg) was solubilized in 1 mL of DMSO. Afterward, the two solutions were mixed and 1 mL of deionized water was added dropwise and left under magnetic stirring for 2 h. This solution was then dialyzed against 1 L of distilled water to remove the free DOX, using a cellulose membrane MWCO 6000–8000 kDa. After 24 h, half of the dialysis bag was lyophilized. The weighted amount of loaded nanoparticles was dissolved in DMSO, and the content of the drug was determined via UV-Vis spectroscopy using a correct blank and a calibration curve.

The drug loading (DL) and encapsulation efficiency (EE) were determined using the following formulas:

% DL = (mass of the DOX loaded into NP/total mass of DOX-loaded NP) × 100(1)

% EE = (mass of the DOX loaded into NP/mass of DOX added initially) × 100(2)

Regarding in vitro release studies, the DOX-loaded nanoparticles were incubated in two aqueous buffers (PBS pH 7.4, citrate–phosphate pH 4.2) under simulated physiological conditions, and half of the solution that had not been lyophilized was placed in a dialysis bag (MWCO 6000–8000 kDa), which was then immersed in 20 mL of buffer and kept under magnetic stirring at 37 °C. For measuring the amount of the drug released, 1.5 mL aliquots were taken out from the releasing medium at scheduled times, and the solution was replaced with an equal volume of a fresh medium. The amount of the released DOX was carried out by absorption spectrometry at λ_max_ (480 nm) using a UV-Vis spectrophotometer [36].

## 3. Results and Discussion

### 3.1. PGGA Esterification

**Figure 1:**
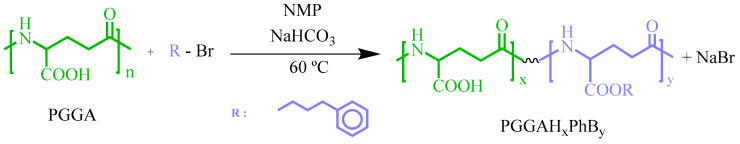
Synthesis of PGGAH_x_PhB_y_ copolymers via the esterification of PGGA with 4-phenyl-butyl bromide.

Esterification of PGGA with 4-phenyl-butyl bromide was carried out according to the method previously reported by Kubota et al. [34] (Figure 1). By varying the concentrations of PGGA, 4-phenyl-butyl bromide, and NaHCO_3_, PGGAH_x_PhB_y_ copolymers with different degrees of esterification were obtained.

Table 1 displays the ratio of reactants used in the feed and the degree of esterification determined via ^1^H NMR, the yields and average molar masses for all PGGAH_x_PhB_y_ copolymers prepared. The copolymers were recovered at high yields (55–97%), as white-to-yellow powders.

### 3.2. Characterization of Copolymers

The weight-average molecular weight of PGGAH_x_PhB_y_ increased almost continuously with the degree of esterification, and all the copolymers assayed showed dispersities between 1.5 and 1.9 (Table 1).

**Figure 2:**
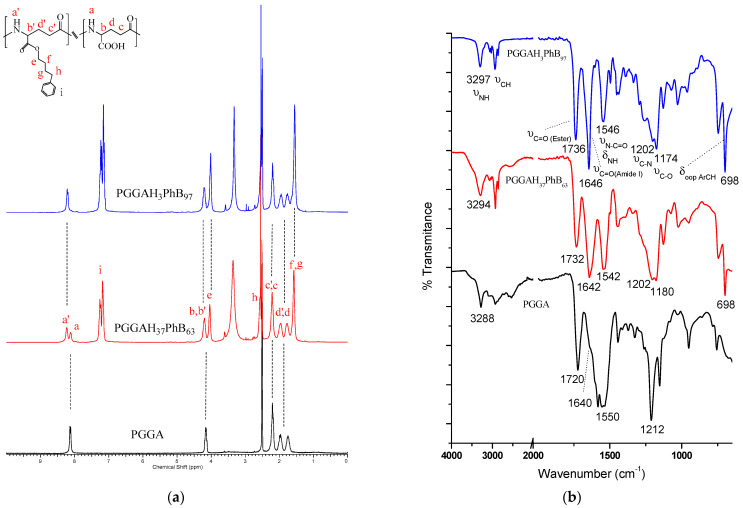
(**a**) ^1^H NMR and (**b**) FTIR spectra of PGGA, and selected PGGAH_x_PhB_y_ copolymers.

^1^H NMR was used to monitor the reaction and determine the degree of esterification achieved (Figure 2a). PGGA displayed four signals from a down- to an up-field shift corresponding to the NH (a, 7.6 ppm), CH (b, 4.2 ppm), α-CH_2_ (c, 2.2 ppm), and β-CH_2_ (d, 1.9 ppm); the last one split due to the presence of an asymmetric center in the repeating unit. On the other hand, copolymers obtained via the esterification of carboxylic groups displayed new peaks at 8.3 ppm (NH, a′), 7.2 (Ar-H, i), 4.0 ppm (OCH_2_, e), 2.6 ppm (CH_2_, h), and 1.6 ppm (CH_2_, f and g). Through the integration of signals due to the α-CH_2_ and aromatic protons, the degree of esterification was calculated.

Figure 2b shows the FTIR spectra of PGGA and two PGGAH_x_PhB_y_ copolymers with an increasing content of the phenyl–butyl side groups. The spectrum of PGGA shows a band centered at 3288 cm^−1^ corresponding to the NH stretching vibration of the amide group. The peak at 1720 cm^−1^ is associated with the stretching vibration of the carbonyl of the COOH side groups, and a small shoulder at 1640 cm^−1^ is due to the stretching vibration of the CO amide group (amide I). When PGGA is partially esterified, it can be observed that the signal of the carbonyl group shifts to higher frequencies, appearing at 1732 and 1736 cm^−1^ for PGGAH_37_PhB_63_ and PGGAH_3_PhB_97_, respectively. This displacement is mainly caused by a reduction in intermolecular hydrogen bonding interactions. Additionally, the appearance of a new peak at 1174–1180 cm^−1^ can be clearly observed on partially esterified PGGA, and was correlated with the C–O stretching vibration. On the other hand, the presence of aromatic groups can be easily identified by the absorption bands at 3027 cm^−1^ corresponding to the Ar–H stretching vibrations, and at 746 and 698 cm^−1^ corresponding to out-of-plane Ar–H bending vibrations. It can be concluded that FTIR spectroscopy is a complementary technique to ^1^H NMR, that allows for the determination of the degree of esterification of the copolymers obtained, at least qualitatively.

The thermal stability of PGGA and PGGAH_x_PhB_y_ copolymers was evaluated via TGA, and data collected from these thermograms are collated in Table 2. The weight loss, concomitant to degradation, occurs between 200 °C and 330 °C. The residual material left at the end of the test decreases in most PGGAH_x_PhB_y_ derivatives with a higher degree of modification.

**Figure 3:**
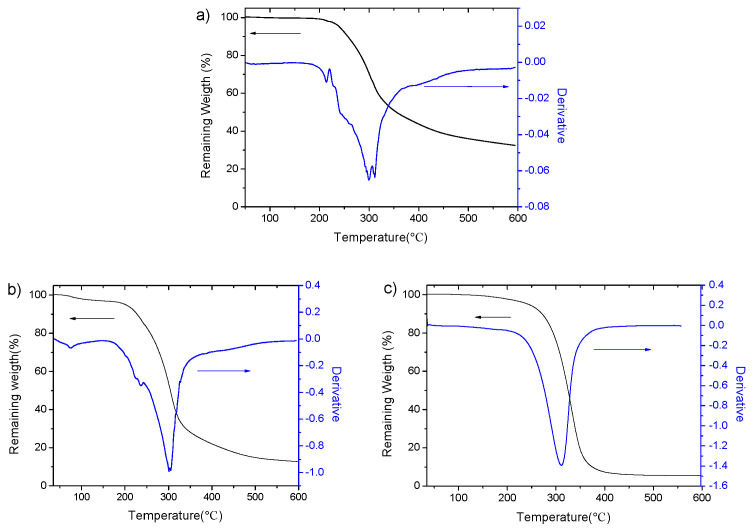
TGA traces recordings of (**a**) PGGA, (**b**) PGGAH_54_PhB_46_, and (**c**) PGGAH_3_PhB_97_, and their corresponding derivative curves. Arrows indicate the vertical scale of each trace.

As can be observed, the decomposition process for most copolymers involves multi-step weight losses, with the main decomposition step taking place between 294 and 332 °C. As an example, the thermal behaviors of PGGA, PGGAH_54_PHB_46_, and PGGAH_3_PhB_97_ are displayed in Figure 3.

### 3.3. Preparation, Characterization, Morphology, and Stability of PGGAH_x_PhB_y_ Nanoparticles over Time

Partial and almost-full modification of PGGA via the esterification of the carboxylate side groups resulted in amphiphilic copolymers that were able to form self-assembled nanostructures. As can be observed in Table 3, all copolymers were able to form particles of nanometric sizes using both nanoprecipitation and nanoemulsion methods. Nanoparticles with average diameters below 200 nm and good polydispersities were obtained for copolymers with intermediate compositions or copolymers with a high degree of esterification. Comparing both methods, it was observed that nanoparticles of smaller sizes could be obtained by nanoprecipitation. On the other hand, all nanoparticles displayed a negative ζ-potential attributed to the remaining carboxylic side groups that were placed at the surface of the nanoparticles, and were ionized at the neutral pH of the water used for the dialysis.

**Figure 4:**
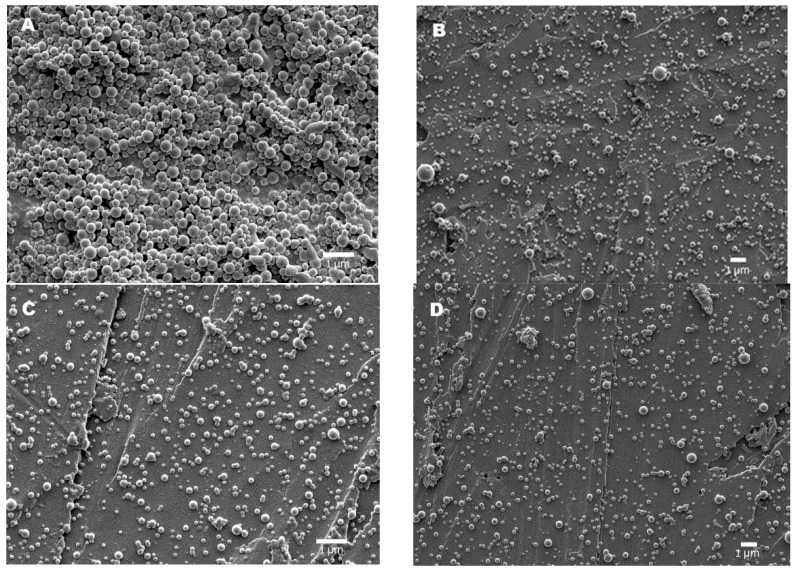
SEM image of nanoparticles from (**A**) PGGAH_54_PhB_46_ via nanoprecipitation, and (**B**) PGGAH_37_PhB_63_, (**C**) PGGAH_25_PhB_75_, and (**D**) PGGAH_3_PhB_97_ via nanoemulsion.

Assays to determine their morphology were performed via SEM. As shown in Figure 4, all particles displayed spherical shapes and nanometric sizes, having average hydrodynamic diameters between 156 and 234 nm (Figure S1).

**Figure 5:**
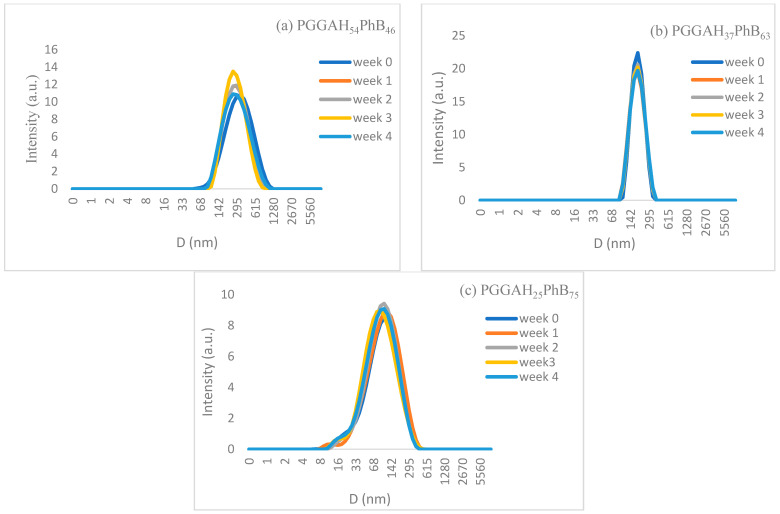
Evolution of DLS curves with the storage time of the nanoparticles obtained from (**a**) PGGAH_54_PhB_46_, (**b**) PGGAH_37_PhB_63_, and (**c**) PGGAH_25_PhB_75_.

The stability of PGGAH_x_PhB_y_ nanoparticles in solution was assessed, maintaining the dispersions over a 4-week period at low temperatures (2–4 °C). As representative examples, three copolymer compositions were assayed, and their stability remained almost unaltered. No precipitation was observed in any sample and the average diameter, as well as polydispersities, remained very stable for this period of time (Table 4 and Figure 5). This good stability can be caused by the small sizes and the high surface charges that the nanoparticles present, which prevents their agglomeration. As can be observed, all nanoparticles displayed high negative ζ-potential values over the period of storage. Only PGGAH_54_PhB_46_ nanoparticles displayed a small reduction in their average diameter after 4 weeks of storage. This striking behavior could be caused by a compaction of the nanoparticles, favored by the hydrophobic interactions and the temperature used during storage.

### 3.4. Doxorubicin Loading and Encapsulation Efficiency

Doxorubicin (DOX) is an outstanding amphiphilic drug that is commonly used in cancer treatment [37]. In order to encapsulate DOX in nanoparticles, the DOX·ClH was previously converted into DOX, and then added to the initial copolymer solution. Nanoparticles were produced by the nanoprecipitation method, and the hydrophobic drug was then encapsulated [38]. In order to check the effect of the copolymer composition on the drug loading and encapsulation efficiency, nanoparticles were prepared from four different copolymers, covering all degrees of esterification (Table 5).

When DOX is used as the hydrochloric acid salt (DOX·HCl), the electrostatic interactions between the drug and the polymer with carboxylic groups maintain the drug at the surface of the nanoparticle. However, these interactions can be easily broken by small changes in the pH or ionic strength [39,40,41,42]. In this work, DOX was transformed in its neutral form through the addition of TEA, allowing for the entering into the core of the nanoparticle. As shown in Table 5, after loading the drug, it was observed that the ζ-potential increased, indicating that there was no neutralization of the surface charge of the nanoparticle; the drug is actually enclosed in the hydrophobic core of the nanoparticle created by the phenyl–butyl side groups grafted in the PGGA. As can be observed, the PGGAH_54_PhB_46_ copolymer displayed higher ζ-potential values than PGGAH_70_PhB_30_ in both loaded and unloaded nanoparticles. Although the number of carboxylate groups is lower in the former copolymer, it seems that it could self-assemble better, exposing greater amounts of carboxylate groups outside the nanoparticles. On the other hand, after loading with DOX, the nanoparticles obtained from PGGAH_89_PhB_11_ displayed micrometric average diameters. It seems that the low content of hydrophobic groups in this copolymer requires a higher number of polymeric chains to stabilize the drug inside the particle.

**Figure 6:**
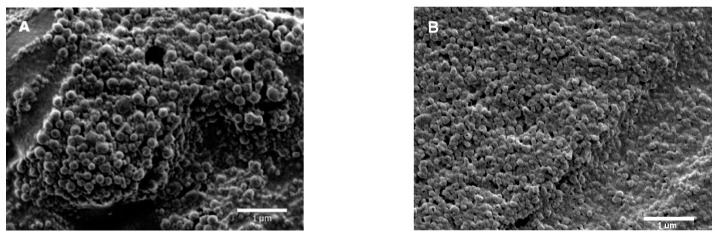
SEM image of DOX-loaded nanoparticles obtained from the (**A**) PGGAH_54_PhB_46_ and (**B**) PGGAH_70_PhB_30_ copolymers.

The amount of drug used in all four samples was the same and equal to 2 ± 0.2 mg, but according to DL- and EE-obtained values, (Table 5), the maximum EE of DOX under neutral conditions after one day of incubation was 46%, obtained for the PGGAH_54_PhB_46_ copolymer. SEM images were collected in order to determine the morphology of the nanoparticles obtained. Quasy-spherical structures corresponding to the nanoparticles loaded with DOX, with average hydrodynamic diameters between 165 and 175 nm, were observed, verifying that the morphology is maintained in relation to the nanoparticles not loaded with DOX (Figure 6 and Figure S2).

### 3.5. In Vitro Drug Release Behavior of NPs

**Figure 7:**
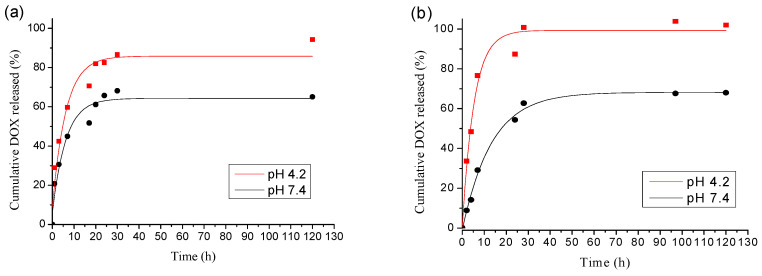
In vitro DOX release from NPs at pH 7.4 and pH 4.2: (**a**) PGGAH_70_PhB_30_ and (**b**) PGGAH_54_PhB_46._

Considering that the fast release in the body minimizes the effects of drugs and has an adverse effect on organs [43,44,45], one of the purposes of this research is to study the effect of PGGA esterification on the drug release behavior of nanoparticles. PBS, with a pH of 7.4 (mimicking the pH of a normal human blood) and a citrate–phosphate pH of 4.2 (lysosomal pH), were used to study the release of DOX in NP. The release curves obtained from the nanoparticles prepared with the two representative PGGAH_x_PhB_y_ copolymers are shown in Figure 7.

Nanoparticles prepared from the PGGAH_70_PhB_30_ and PGGAH_54_PhB_46_ copolymers release 38% and 21% DOX, respectively, in the first 5 h at a pH of 7.4. As expected, the release is more sustained for nanoparticles obtained from the copolymer with a higher content of phenyl–butyl side groups, since it will have a greater ability to interact with the DOX hydrophobic drug. Surprisingly, the trend was reversed in the release profile at a pH of 4.2, and the release rate was greater for the copolymer with the higher degree of esterification. The in vitro maximum release of DOX was almost 100% for PGGAH_54_PhB_46_ and 95% for PGGAH_70_PhB_30_. A combined effect of a greater destabilization of the nanoparticle, due to the partial loss of the surface charge and the protonation of the amino groups of the DOX, may be the cause of this behavior. Contrastingly, the higher release rate noticed in acid media has also been observed in the micelles of other hydrophobically modified polypeptides, such as poly(α,β-aspartic acid) and PEG-grafted poly(α-glutamic acid), and attributed to the formation of agglomerates in the latter case, that release the cargo and are caused by a reduction in the repulsion charges [46,47]. In our case, we believe that water can swell the nanoparticle and, in the case of having an acid release medium, the amino group of the DOX can become protonated, thus increasing its solubility, allowing its faster diffusion from the nanoparticle to the medium.

Drug release profiles have been fitted to different kinetic models (zero-order, first-order, Higuchi, and Korsmeyer–Peppas models, Table S1) [48]. It was observed that the best fit of the release profile was achieved with the Korsmeyer–Peppas model. Since *n* < 0.45 for nanoparticles obtained from PGGAH_70_PhB_30_ at both pH conditions, a Fickian diffusion mechanism of drug, outward the nanoparticles, was suggested. On the other hand, values of 0.74 and 0.65 *n* were obtained at pHs 7.4 and 4.2, respectively, for the DOX-loaded nanoparticles made of PGGAH_54_PhB_46_, indicating an anomalous (non-Fickian) diffusion behavior for this copolymer composition.

Nanoparticles sensitive to pH, such as those obtained here, can release drugs quickly at the tumor site and very slowly in the peripheral circulation [49,50,51,52,53,54]. The optimization of nanoparticles through PEGylation and adding targeting ligands is a work that is planned to be carried out by us in the near future.

## 4. Conclusions

Amphiphilic copolymers have been obtained via the partial esterification of bacterial poly(γ-glutamic acid) with 4-phenyl-butyl bromide. These copolymers were able to self-assemble into spherical nanoparticles with average diameters of around 200 nm, using nanoprecipitation and nanoemulsion methods. The hydrophobic core was composed of repeating units containing the phenyl–butyl ester groups and the hydrophilic shell, composed of repeating units with unreacted carboxylate groups. These nanoparticles were able to encapsulate Doxorubicin at a high encapsulation efficiency, and release it faster at acidic pHs. These results indicate that the modified PGGA copolymers can be used for preparing nanoparticles that act as anti-cancer drug carriers of hydrophobic drugs, such as DOX.

## Figures and Tables

**Table 1:** Compositions and molecular weights of the PGGAH_x_PhB_y_ copolymers.

Copolymer	Feed ^1^	Esterification Degree ^2^	Yield	*M*_w_ ^3^	Ð
	(mg:mL:mg)	(%)	(%)	(g·mol^−1^)	
PGGAH_95_PhB_5_	500:0.029:340	4.6	92	-	-
PGGAH_89_PhB_11_	500:0.135:340	11	55	-	-
PGGAH_70_PhB_30_	500:0.270:340	29.9	75	-	-
PGGAH_54_PhB_46_	500:0.810:340	46.2	77	23,550	1.9
PGGAH_37_PhB_63_	500:0.675:639	63	90	22,900	1.9
PGGAH_25_PhB_75_	500:1.080:1022	75	94	33,200	1.7
PGGAH_3_PhB_97_	500:1.282:1210	97	97	34,550	1.9

^1^ PGGA: 4-phenyl-butyl bromide–NaHCO_3_ ratios. ^2^ Degree of esterification of PGGA calculated via ^1^H-NMR; ^3^ Weight-average molecular weight and dispersity determined via GPC (PGGAH_x_PhB_y_ copolymers with a low degree of esterification were insoluble in HFIP).

**Table 2:** Thermal stability of PGGA and PGGAH_x_PhB_y_ copolymers.

Copolymers	T_d_ ^1^ (°C)	T_d1_/T_d2_/T_d3_^2^ (°C)	R_W_^3^ (%)
PGGA	282	**299**/311	31.3
PGGAH_95_PhB_5_	256	**311**/245/216	28.5
PGGAH_89_PhB_11_	252	**309**/216	27.5
PGGAH_70_PhB_30_	235	**294**/354/255	26.1
PGGAH_54_PhB_46_	233	**304**/308/240	12.9
PGGAH_37_PhB_63_	237	**322**/205	8.9
PGGAH_25_PhB_75_	268	**331**	4.4
PGGAH_3_PhB_97_	274	**332**	5.4

^1^ Onset decomposition temperature measured at 10% of loss of the initial weight. ^2^ Maximum rate decomposition temperature. In bold the temperature of main decomposition step. ^3^ Remaining weight at 600 °C.

**Table 3:** Characterization of nanoparticles by DLS.

Copolymers	Nanoprecipitation			Nanoemulsion		
	D	Pd.I ^1^	ζ-Pot	D	Pd.I ^1^	ζ-Pot
	(nm)		(mV)	(nm)		(mV)
PGGAH_95_PhB_5_	374 ± 8.3	0.15 ± 0.03	−48.4 ± 0.8	-	-	-
PGGAH_89_PhB_11_	296 ± 9.8	0.17 ± 0.03	−49.5 ± 1.1	-	-	-
PGGAH_70_PhB_30_	180 ± 2.1	0.29 ± 0.02	−31.7 ± 1.3	-	-	-
PGGAH_54_PhB_46_	297 ± 1.5	0.22 ± 0.01	−40.6 ± 1.0	-	-	-
PGGAH_37_PhB_63_	184 ± 0.7	0.04 ± 0.01	−35.6 ± 0.1	245 ± 4.2	0.24 ± 0.02	−40.0 ± 1.4
PGGAH_25_PhB_75_	89 ± 1.8	0.40 ± 0.01	−35.5 ± 0.6	175 ± 1.6	0.16 ± 0.01	−37.1 ± 1.6
PGGAH_3_PhB_97_	146 ± 2.0	0.38 ± 0.01	−30.1 ± 0.4	157 ± 4.6	0.10 ± 0.01	−13.1 ± 1.2

^1^ Polydispersity index.

**Table 4:** Size distribution and ζ-pot of PGGAH_x_PhB_y_ nanoparticles, with time stored at 2–4 °C.

Copolymers	Week 1			Week 2			Week 3			Week 4		
	D (nm)	Pd.I	ζ-Pot (mV)	D (nm)	Pd.I	ζ-Pot (mV)	D (nm)	Pd.I	ζ-Pot (mV)	D (nm)	Pd.I	ζ-Pot (mV)
PGGAH_54_PhB_46_	258 ± 1.3	0.16 ± 0.00	−36.9 ± 2.4	261 ± 0.5	0.16 ± 0.00	−35.9 ± 0.9	254 ± 3.6	0.13 ± 0.01	−36.8 ± 0.9	258 ± 0.9	0.18 ± 0.00	−35.0 ± 0.4
PGGAH_37_PhB_63_	186 ± 0.6	0.04 ± 0.04	−35.0 ± 1.5	184 ± 1.7	0.03 ± 0.01	−36.9 ± 0.5	184 ± 1.7	0.08 ± 0.00	−40.7 ± 1.0	183 ± 1.0	0.03 ± 0.02	−41.6 ± 3.4
PGGAH_25_PhB_75_	86 ± 0.4	0.28 ± 0.00	−33.1 ± 9.1	82 ± 1.1	0.29 ± 0.00	−30.4 ± 7.9	76 ± 1.7	0.27 ± 0.00	−29.8 ± 0.7	76 ± 0.3	0.27 ± 0.00	−20.5 ± 2.1

**Table 5:** Nanoparticle properties obtained from different PGGAH_x_PhB_y_, drug loading and encapsulation efficiency.

Copolymers	Unloaded			Loaded Doxorubicin		
	D (nm)	ζ-Pot (mV)	D (nm)	ζ-Pot (mV)	EE% ^1^	DL% ^2^
PGGAH_89_PhB_11_	296 ± 9.8	−49.5 ± 1.1	1594 ± 57.7	−49.6 ± 12.0	27	6.0
PGGAH_70_PhB_30_	180 ± 2.0	−31.7 ± 1.3	216 ± 3.3	−42.4 ± 2.9	36	7.1
PGGAH_54_PhB_46_	163 ± 1.5	−40.6 ± 1.0	170 ± 4.9	−47.4 ± 1.7	46	10.1
PGGAH_3_PhB_97_	146 ± 2.0	−30.1 ± 0.4	102 ± 0.2	−40.3 ± 2.0	40	8.4

^1^ Encapsulation efficiency. ^2^ Drug loading.

## Data Availability

Data is contained within the article.

## Supplementary Materials

The following supporting information can be downloaded at: https://www.mdpi.com/article/10.3390/pharmaceutics15051377/s1, Figure S1: Size distributions and average particle diameters (Υ) of PGGAH_x_PhB_y_ copolymers determined by SEM; Figure S2: Size distributions and average particle diameters (Υ) of DOX-loaded PGGAH_x_PhB_y_ copolymers determined by SEM; Table S1: Comparison of mathematical models of the 24 h release profiles of Doxorubicin from PGGAH_x_PhB_y_ copolymers at different pHs.
