# Supplementary material for: Hydrophobic Modification of Poly(γ-glutamic acid) by Grafting 4-Phenyl-butyl Side Groups for the Encapsulation and Release of Doxorubicin
Source: Pharmaceutics. 2023 Apr 29;15(5):1377. doi: 10.3390/pharmaceutics15051377 (PMC10222848; doi:10.3390/pharmaceutics15051377)
Supplement: Supplementary file 1 [file pharmaceutics-15-01377-s001.zip › pharmaceutics-2284289-supplementary.pdf]

pharmaceutics

MDPI

Supplementary Information: Hydrophobic Modification of Poly(γ-Glutamic Acid) by Grafting 4-Phenyl-butyl Side Groups for the Encapsulation and Release of Doxorubicin

Porochista Dorost [1], Montserrat García-Alvarez [1,*] and Antxon Martínez de Ilarduya [1,*]

[1] Departament d´Enginyeria Química, Universitat Politècnica de Catalunya, ETSEIB, Diagonal 647, 08028 Barcelona, Spain

* Correspondence: montserrat.garcia@upc.edu, antxon.martinez.de.ilarduia@upc.edu

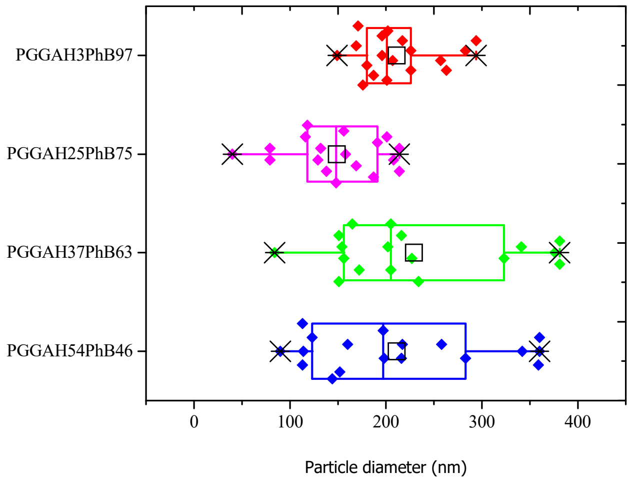

Figure S1. Size distributions and average particle diameters (□) of PGGAH$_x$PhB$_y$ copolymers determined via SEM.

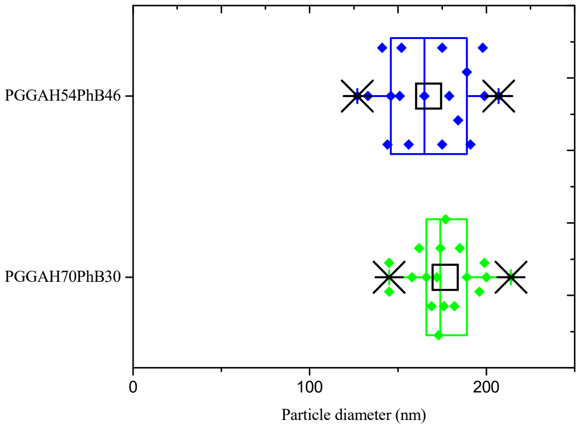

Figure S2. Size distributions and average particle diameters (□) of DOX-loaded PGGAH$_x$PhB$_y$ copolymers determined via SEM.

Table S1. Comparision of mathematical models of the 24-h release profiles of Doxorubicin from $PGGAH_xPhB_y$ copolymers at different pHs.

Copolymer	pH	Zero-order model		First-order model		Higuchi model		Korsmeyer–Peppas model[1]		
		K	r^2	*K*	r^2	*K*	r^2	*K*	*n*	r^2
$PGGAH_{70}PhB_{30}$	7.4	0.022	0.81	-0.038	0.91	0.123	0.96	0.211	0.35	0.98
	4.2	0.028	0.78	-0.066	0.93	0.159	0.95	0.295	0.33	0.99
$PGGAH_{54}PhB_{46}$	7.4	0.021	0.92	-0.032	0.97	0.132	0.97	0.055	0.74	0.95
	4.2	0.029	0.52	-0.079	0.78	0.145	0.69	0.208	0.65	0.97

[1] Only data from the first 60% of DOX release were used to statistically analyze the results.
